# Vitamin E Ameliorates the Decremental Effect of Paraquat on Cardiomyocyte Contractility in Rats

**DOI:** 10.1371/journal.pone.0057651

**Published:** 2013-03-18

**Authors:** Mohamed Abdelmonem Fahim, Frank Christopher Howarth, Abderrahim Nemmar, Mohamed Anwar Qureshi, Mohamed Shafiullah, Petrilla Jayaprakash, Mohamed Yousif Hasan

**Affiliations:** 1 Department of Physiology, Faculty of Medicine, United Arab Emirates University, Al Ain, United Arab Emirates; 2 Department of Pharmacology, Faculty of Medicine, United Arab Emirates University, Al Ain, United Arab Emirates; Temple University, United States of America

## Abstract

**Background:**

Exposure to pesticides and industrial toxins are implicated in cardiovascular disease. Paraquat (PAR) is a toxic chemical widely used as an herbicide in developing countries and described as a major suicide agent. The hypothesis tested here is that PAR induced myocardial dysfunction may be attributed to altered mechanisms of Ca^2+^ transport which are in turn possibly linked to oxidative stress. The mechanisms of PAR induced myocardial dysfunction and the impact of antioxidant protection was investigated in rat ventricular myocytes.

**Methodology:**

Forty adult male Wistar rats were divided into 4 groups receiving the following daily intraperitoneal injections for 3 weeks: Group 1 PAR (10 mg/kg), Control Group 2 saline, Group 3 vitamin E (100 mg/kg) and Group 4 PAR (10 mg/kg) and vitamin E (100 mg/kg). Ventricular action potentials were measured in isolated perfused heart, shortening and intracellular Ca^2+^ in electrically stimulated ventricular myocytes by video edge detection and fluorescence photometry techniques, and superoxide dismutase (SOD) and catalase (CAT) levels in heart tissue.

**Principal Findings:**

Spontaneous heart rate, resting cell length, time to peak (TPK) and time to half (THALF) relaxation of myocyte shortening were unaltered. Amplitude of shortening was significantly reduced in PAR treated rats (4.99±0.26%) and was normalized by vitamin E (7.46±0.44%) compared to controls (7.87±0.52%). PAR significantly increased myocytes resting intracellular Ca^2+^ whilst TPK and THALF decay and amplitude of the Ca^2+^ transient were unaltered. The fura-2–cell length trajectory during the relaxation of the twitch contraction was significantly altered in myocytes from PAR treated rats compared to controls suggesting altered myofilament sensitivity to Ca^2+^ as it was normalized by vitamin E treatment. A significant increase in SOD and CAT activities was observed in both PAR and vitamin E plus PAR groups.

**Conclusions:**

PAR exposure compromised rats heart function and ameliorated by vitamin E treatment.

## Introduction

Cardiovascular disease is the major cause of premature mortality in both the developed and developing world. It is noteworthy that a number of risk factors which are associated with cardiovascular disease may be linked, at least in part, by oxidative stress. Oxidative stress can lead to dysfunction in endothelial cells, monocytes and vascular smooth muscle cells as well as mitochondrial damage [1]–[2]. Oxidative stress and DNA damage are induced by oxidized low density lipoproteins and by diet-induced hypercholesterolemia and this has the potential to contribute to dysfunction of endothelial cells, vascular smooth muscle cells, T lymphocytes and macrophages [3]–[5].

The maintenance of physiological cardiac structure and function is essentially dependent on oxidant balance. Mitochondrial respiration, enzymatic reactions, and inflammatory response may play a collective role in balancing the production of reactive oxygen species (ROS), and endogenous antioxidant defense system composed of antioxidant molecules and enzymes to counteract the damaging effects of ROS by converting more reactive species to less reactive and less damaging forms [6]–[8]. The antioxidant reserve often becomes inadequate under pathological conditions, leading to ROS accumulation-triggered oxidative stress and myocardial geometric and functional defects [7]. Although a number of mechanisms have been postulated for oxidative stress-induced myopathic changes, including mitochondrial damage, defective mechanimsms of Ca^2+^ transport, oxidative modification of essential cardiac contractile proteins, and direct cardiac toxicity of ROS [7]–[9], the mechanisms of which underlie “oxidative cardiomyopathy” have not been clearly elucidated.

Epidemiological studies have revealed that chronic exposure to pesticides such as paraquat (PAR) and other environmental toxins are involved in the progression of Parkinson's disease [10]. For example, a dose-dependent lifetime cumulative exposure relationship of PAR (1,1′-dimethyl-4,4′- bipyridinium dichloride, a quaternary ammonium herbicide commonly used as a weed controller) and increased risk for Parkinson's disease has been reported [11]–[13]. This could be due to the fact that the chemical structure of PAR resembles that of MPTP (1-methyl-4-phenyl-1,2,3,6-tetrahydropyridine), a neurotoxin known to induce Parkinsonism in humans and experimental animals [12], [14]. Furthermore, administration of PAR to mice causes selective degeneration of dopaminergic neurons in the substantia nigra, thus reproducing one of the primary pathological features of Parkinson's disease [15], [16].

Parallel work in rodents has demonstrated that administration or accidental ingestion of PAR causes an extremely high fatality rate (30–70%) [17], [18]. PAR catalyzes the formation of ROS. Within aerobically living cells, ROS are continuously produced to carry out biological reactions. Overproduction, however, can damage cell membranes through the peroxidation of membrane polyunsaturated fatty acids. The mechanisms of PAR toxicity involve generation of ROS leading to oxidative stress which is an imbalanced state between the formations of ROS and scavenging by antioxidant. The ROS reacts with polyunsaturated fatty acids and produces toxic aldehyde metabolites which are the principle end products of lipid peroxidation. Among various antioxidants, SOD and catalase constitute the primary enzymatic defence system [6]–[8]. PAR, therefore, is considered to be a highly toxic pro-oxidant that causes multiorgan failure including that of the heart via generation of ROS. PAR has been shown to overtly compromise myocardial survival and contractile function en route to cardiopulmonary failure [19]–[21]. In the present study, the mechanism(s) of action behind PAR induced myocardial dysfunction and the impact of antioxidant protection has been investigated in rat ventricular myocytes.

## Methods

### Animal Ethics

Animal care was conducted in accordance with the United States Public Health Service Guide for the Care and Use of Laboratory Animals. In order to safeguard animal welfare and avoid suffering such as the extent of failure to cope with the PAR treatment, daily monitoring of animal health were conducted by a specialist veterinarian. Approval for this project was obtained from the Animal Ethics Committee, Faculty of Medicine & Health Sciences, United Arab Emirates University.

### Experimental protocol

Forty adult male Wistar rats were divided into 4 groups. Animals received daily intraperitoneal injections for 3 weeks as follows; Group 1 received PAR (10 mg/kg), Group 2 served as controls and received saline, Group 3 received vitamin E (100 mg/kg) and Group 4 received PAR (10 mg/kg) and vitamin E (100 mg/kg). Animals were housed in polypropylene cages with a controlled light and dark cycle of 12 hours each at 24–26°C. Food and water were available *ad libitum*. Repeated administration of small doses (10 mg/kg) of PAR was chosen to maintain a subthreshold dose of PAR and simulate the chronic farmers exposure.

### Measurement of heart rate and action potential duration

Action potentials and heart rate were recorded according to previously described techniques [22]. Animals were humanely sacrificed by the use of a guillotine. Hearts were removed rapidly, mounted in Langendorff mode and perfused at a constant flow of 8 ml.g heart^−1^.min^−1^ at physiological temperature (36–37°C) with normal Tyrode solution containing in mmol/l: 140 NaCl, 5 KCl, 1 MgCl_2_, 10 glucose, 5 HEPES, 1.8 CaCl_2_ and adjusted to pH 7.4 with NaOH and continuously bubbled with oxygen. When contraction of the heart had stabilized extracellular action potentials were recorded from the left ventricle in spontaneously beating hearts with a purpose built extracellular suction electrode. Signals from the electrode were collected at 1000 Hz, amplified (ML136 Bioamp, AD Instruments, Australia) and conveyed via a Powerlab 4/30 (ML 866, AD Instruments, Australia) to a PC. Time from threshold of action potential to 50% (APD50) and 70% (APD70) repolarization of action potentials were measured. Data were acquired and analyzed with ADInstruments Labchart 7 Pro v 7.0.3 (AD Instruments, Australia).

### Ventricular myocyte isolation

Myocytes were isolated according to previously described techniques [22]. After sacrifice hearts were removed rapidly and mounted for retrograde perfusion according to the Langendorff method. Hearts were perfused at a constant flow of 8 ml.g heart^−1^.min^−1^ at 36–37°C with cell isolation solution containing in mmol/l: 130.0 NaCl, 5.4 KCl, 1.4 MgCl_2_, 0.75 CaCl_2_, 0.4 NaH_2_PO_4_, 5.0 HEPES, 10.0 glucose, 20.0 taurine and 10.0 creatine (pH 7.3). Perfusion flow rate was adjusted to allow for differences in heart weight between animals. When the heart had stabilized perfusion was continued for 4 min with Ca^2+^-free cell isolation solution containing 0.1 mmol/l EGTA, and then for 6 min with cell isolation solution containing 0.05 mmol/l Ca^2+^, 0.75 mg/ml type 1 collagenase (Worthington Biochemical Corp, USA) and 0.075 mg/ml type XIV protease (Sigma, Germany). Left ventricle tissue was excised from the heart, minced and gently shaken in collagenase-containing isolation solution supplemented with 1% BSA. Cells were filtered from this solution at 4 min intervals and resuspended in cell isolation solution containing 0.75 mmol/l Ca^2+^.

### Measurement of ventricular myocyte shortening

Ventricular myocyte shortening was measured according to previously described techniques [22]. Cells were allowed to settle on the glass bottom of a Perspex chamber mounted on the stage of an inverted Axiovert 35 microscope (Zeiss, Germany). Cells were superfused (3–5 ml/min) with normal Tyrode containing the following in mmol/l: 140.0 NaCl, 5.0 KCl, 1.0 MgCl_2_, 10.0 glucose, 5.0 HEPES, 1.8 CaCl_2_ (pH 7.4). Unloaded myocyte shortening was recorded using a VED-114 video edge detection system (Crystal Biotech, USA). Resting cell length, time to peak (TPK) shortening, time from peak to half (THALF) relaxation of shortening and amplitude of shortening (expressed as a % of resting cell length) were measured in electrically stimulated (1 Hz) myocytes maintained at 35–36°C. Recording of shortening commenced when myocyte contraction was stable. Data were acquired and analyzed with Signal Averager software v 6.37 (Cambridge Electronic Design, UK).

### Measurement of intracellular Ca^2+^

Intracellular Ca^2+^ was measured according to previously described techniques [22]. Myocytes were loaded with the fluorescent indicator fura-2 AM (F-1221, Molecular Probes, USA). In brief, 6.25 µl of a 1.0 mmol/l stock solution of fura-2 AM (dissolved in dimethylsulphoxide) was added to 2.5 ml of cells to give a final fura-2 concentration of 2.5 µmol/l. Myocytes were shaken gently for 10 min at room temperature (24°C). After loading, myocytes were centrifuged, washed with normal Tyrode to remove extracellular fura-2 and then left for 30 min to ensure complete hydrolysis of the intracellular ester. To measure intracellular Ca^2+^ myocytes were alternately illuminated by 340 nm and 380 nm light using a monochromator (Cairn Research, UK) which changed the excitation light every 2 ms. The resulting fluorescence emitted at 510 nm was recorded by a photomultiplier tube and the ratio of the emitted fluorescence at the two excitation wavelengths (340/380 ratio) was calculated to provide an index of intracellular Ca^2+^ concentration. Resting fura-2 ratio, TPK Ca^2+^ transient, time from peak to half (THALF) decay of the Ca^2+^ transient and the amplitude of the Ca^2+^ transient were measured in electrically stimulated (1 Hz) myocytes maintained at 35–36°C. Data were acquired and analyzed with Signal Averager software v 6.37 (Cambridge Electronic Design, UK).

### Measurement of myofilament sensitivity to Ca^2+^

In some cells shortening and Ca^2+^ were measured simultaneously. Myofilament sensitivity to Ca^2+^ was assessed from phase-plane diagrams of fura-2 ratio vs. cell length by measuring the gradient of the fura-2 - cell length trajectory during late relaxation of the twitch contraction according to previously described techniques [23]. The position of the trajectory reflects the relative myofilament response to Ca^2+^ and hence, can be used as a measure of myofilament sensitivity to Ca^2+^ [24].

### Measurement of oxidative stress, lipid peroxidation and antioxidant enzyme activities in heart tissue

In separate rats (n = 6 in each group), following exposure to PAR or saline with or without vitamin E pretreatment, animals were sacrificed with an overdose of sodium pentobarbital. Heart tissues were collected and rinsed with ice-cold PBS (pH 7.4) before homogenization in 0.1 M phosphate buffer pH 7.4 containing 0.15 M KCl, 0.1 mM EDTA, 1 mM DTT and 0.1 mM phenylmethylsulfonylfluoride at 4°C. Homogenate was centrifuged for 10 min at 3000×g to remove cellular debris and supernatants were used for further analysis. Protein content was measured by Bradford's method as described before [1]. The supernatant was stored at −80°C for the determination of SOD and CAT (kits from Cayman Chemical, Ann Arbor, MI, USA) levels [25].

### Statistics

Results were expressed as the mean ± S.E.M. of ‘n’ observations. Statistical comparisons were performed using either the Independent samples t-test or one-way ANOVA followed by Bonferroni corrected t-tests for multiple comparisons, as appropriate. P<0.05 was considered to indicate a significant difference.

## Results

### General characteristics

The weight of the rats were assessed and recorded during and after PAR treatment. No significant changes were observed in weight of PAR treated group (204.0±2.7 g initially and 247.2±2.4 g after three weeks) when compared to the saline treated control group (204.9±3.4 g initially and 257.8±6.3 g after three weeks). No significant changes were observed after vitamin E treatment (210.0±4.6 g initially and 251.3±4.8 g after three weeks) when compared to the saline treated control group (204.9±3.4 g initially and 257.8±6.3 g after three weeks). Also, no significant changes were observed after PAR and vitamin E treatment (207.2±4.2 g initially and 251.6±3.9 g after three weeks) when compared to the saline treated control group (204.9±3.4 g initially and 257.8±6.3 g after three weeks).

### Heart rate and action potential duration

**Figure 1:**
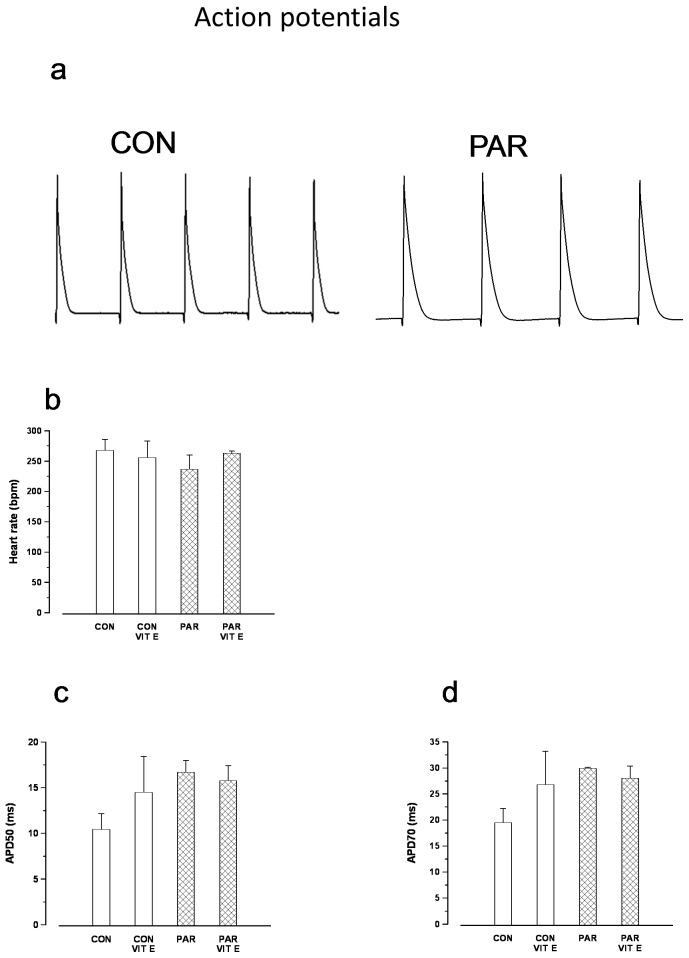
Typical recording of action potentials from hearts of control and PAR treated rats (a). Heart rate (b), APD50 (c) and APD70 (d). Data are mean±SEM, n = 4–5 hearts in each treatment group. CON = Control, PAR = Paraquat, VIT E = Vitamin E.

Typical action potentials recorded from the left ventricle with a suction electrode in the spontaneously beating heart are shown in Figure 1a. Spontaneous heart rate was not significantly (p>0.05) altered in PAR treated animals (237±23 bpm) compared to controls (268±18 bpm) (Figure 1b). APD50 (Figure 1c) and APD70 (Figure 1d) were also not significantly altered in PAR treated animals compared to control animals.

### Myocyte shortening

**Figure 2:**
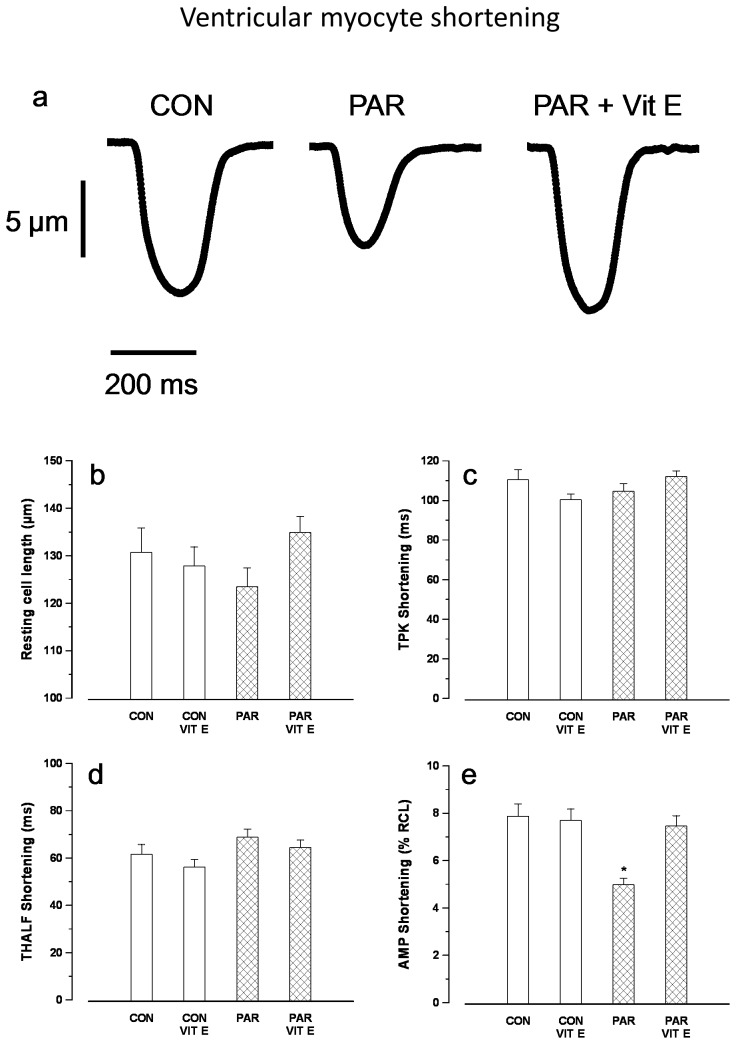
Typical records of shortening in myocytes from control, PAR and PAR+ vitamin E treated rats (a). Resting cell length (b), time to peak (TPK) shortening (c), time to half (THALF) relaxation (d) and amplitude of shortening (e). Data are mean±SEM, n = 16–29 cells from 3 hearts in each treatment group. CON = Control, PAR = Paraquat, VIT E = Vitamin E. *p<0.05 between PAR and CON, CON+VIT E and PAR+VIT E groups.

Typical records of myocyte shortening are shown in Figure 2a. Resting cell length (Figure 2b), TPK shortening (Figure 2c) and THALF relaxation (Figure 2d) were not significantly altered in PAR treated compared to control animals. Amplitude of shortening (Figure 2e) was significantly (p<0.05) reduced in PAR treated (4.99±0.26%) compared to controls (7.87±0.52%). Interestingly, the negative inotropic effects demonstrated in PAR treated animals were not seen in animals that received vitamin E treatment (7.47±0.44%). Amplitude of shortening in PAR + vitamin E (7.46±0.44%) and vitamin E (7.70±0.48%) treated animals were not significantly different (p>0.05) compared to controls.

### Intracellular calcium

**Figure 3:**
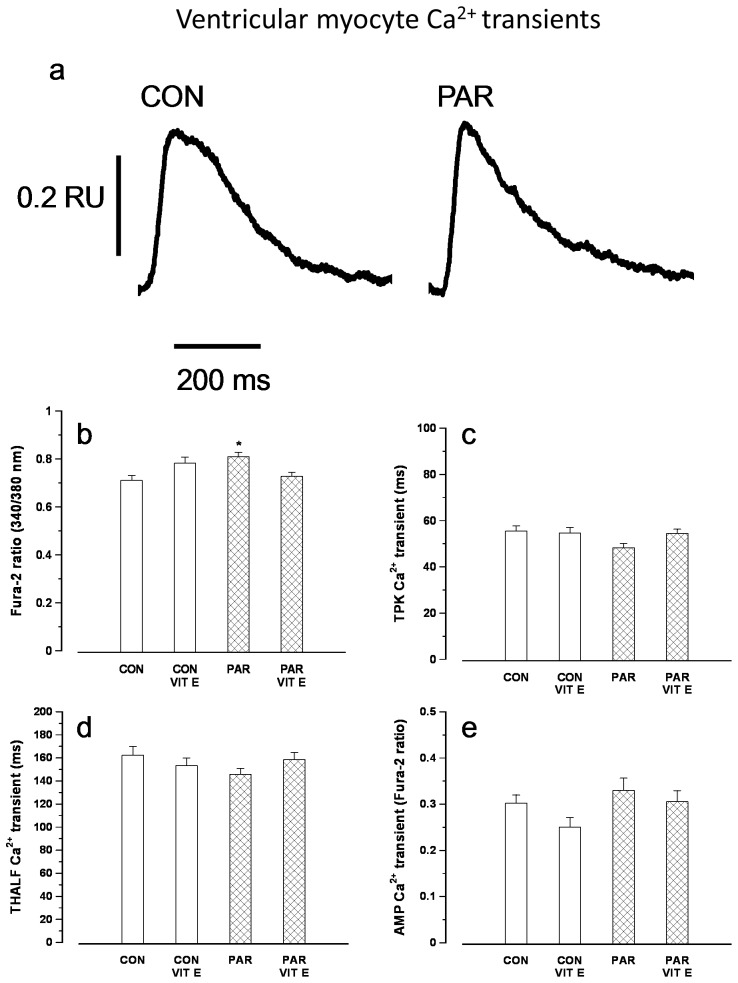
Typical records of Ca^2+^ transients in myocytes from control and PAR treated rats (a). Resting fura-2 ratio (b), time to peak (TPK) Ca^2+^ transient (c), time to half (THALF) decay of the Ca^2+^ transient (d) and amplitude of the Ca^2+^ transient (e). Data are mean±SEM, n = 20–32 cells from 3 hearts in each treatment group. CON = Control, PAR = Paraquat, VIT E = Vitamin E.

Typical records of intracellular Ca^2+^ transients are shown in Figure 3a. Resting intracellular Ca^2+^ was significantly elevated in PAR treated compared to control myocytes (Figure 3b). TPK Ca^2+^ transient (Figure 3c), THALF decay of the Ca^2+^ transient (Figure 3d) and amplitude of the Ca^2+^ transient (Figure 3e) were not significantly altered in myocytes from PAR treated compared to controls. Amplitude of the Ca^2+^ transient in PAR+vitamin E and vitamin E treated animals were not significantly different (p>0.05) compared to controls.

### Measurement of myofilament sensitivity to Ca^2+^

**Figure 4:**
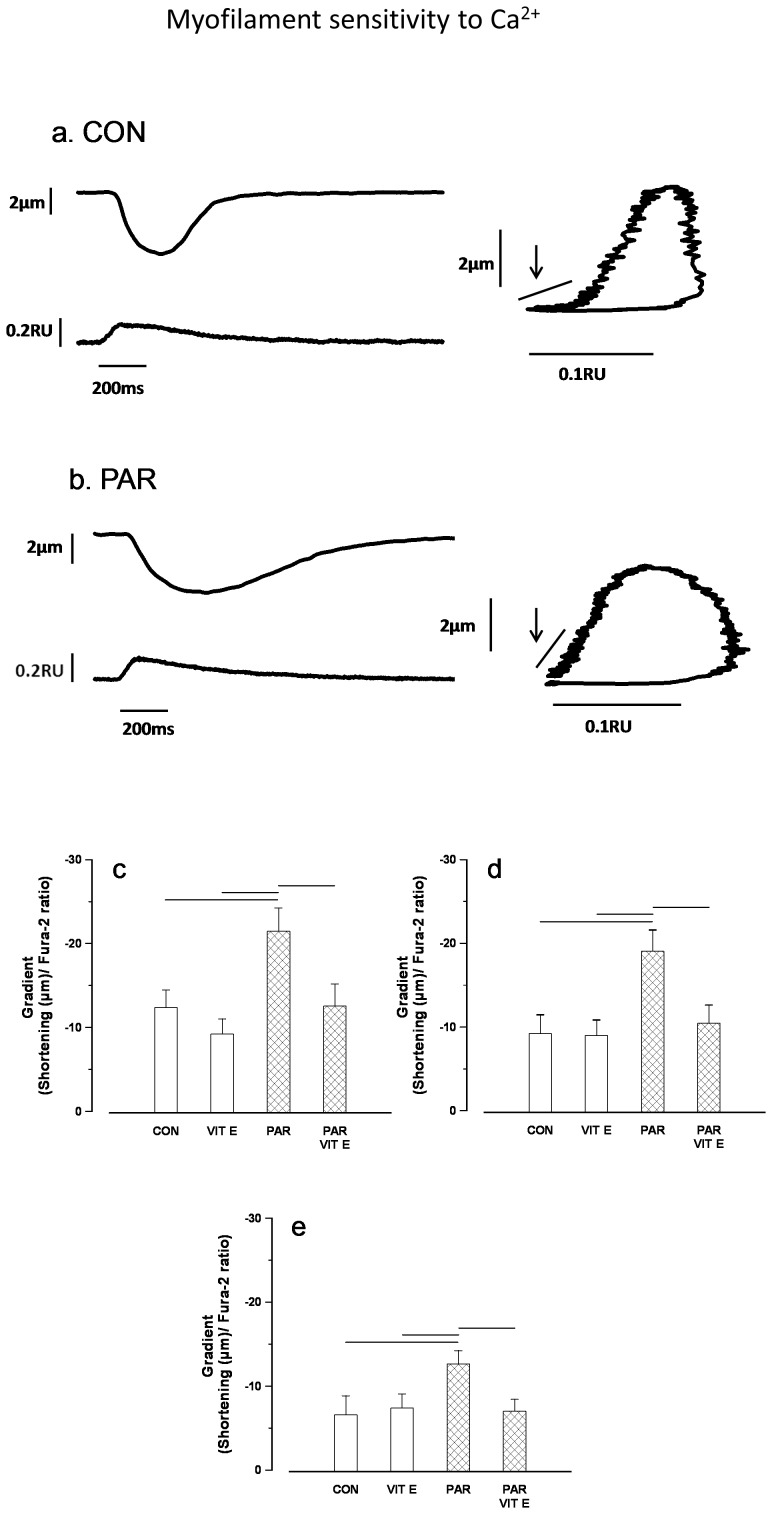
Typical records showing simultaneous recordings of shortening and fura-2 ratio and phase-plane diagrams in control (a) and PAR treated rats (b). Mean gradient of fura-2-cell length trajectory during late relaxation of the twitch contraction during the periods 500–800 (c), 500–700 (d) and 500–600 ms (e). Data are mean ± SEM, n = 19–31 cells from 3 hearts in each treatment group. CON = Control, PAR = Paraquat, VIT E = Vitamin E. Horizontal lines above bars represent P<0.05.

In some experiments myocyte shortening and intracellular Ca^2+^ were recorded simultaneously and typical recordings in ventricular myocytes from PAR treated and control rats are shown in Figure 4a & b (left panel). Typical phase-plane diagrams of fura-2 ratio vs. cell length in ventricular myocytes from PAR treated and control rats are shown in figure 4a & b (right panel). The mean gradient of fura-2-cell length trajectory during late relaxation of the twitch contraction was significantly (p<0.05) altered in myocytes from PAR treated rats at 500–800 (Figure 4c), 500–700 (Figure 4d) and 500–600 ms (Figure 4e). The mean gradient of fura-2-cell length trajectory in PAR+vitamin E and vitamin E treated animals were not significantly different (p>0.05) compared to controls.

### Catalase and superoxide dismutase levels in heart tissue

**Figure 5:**
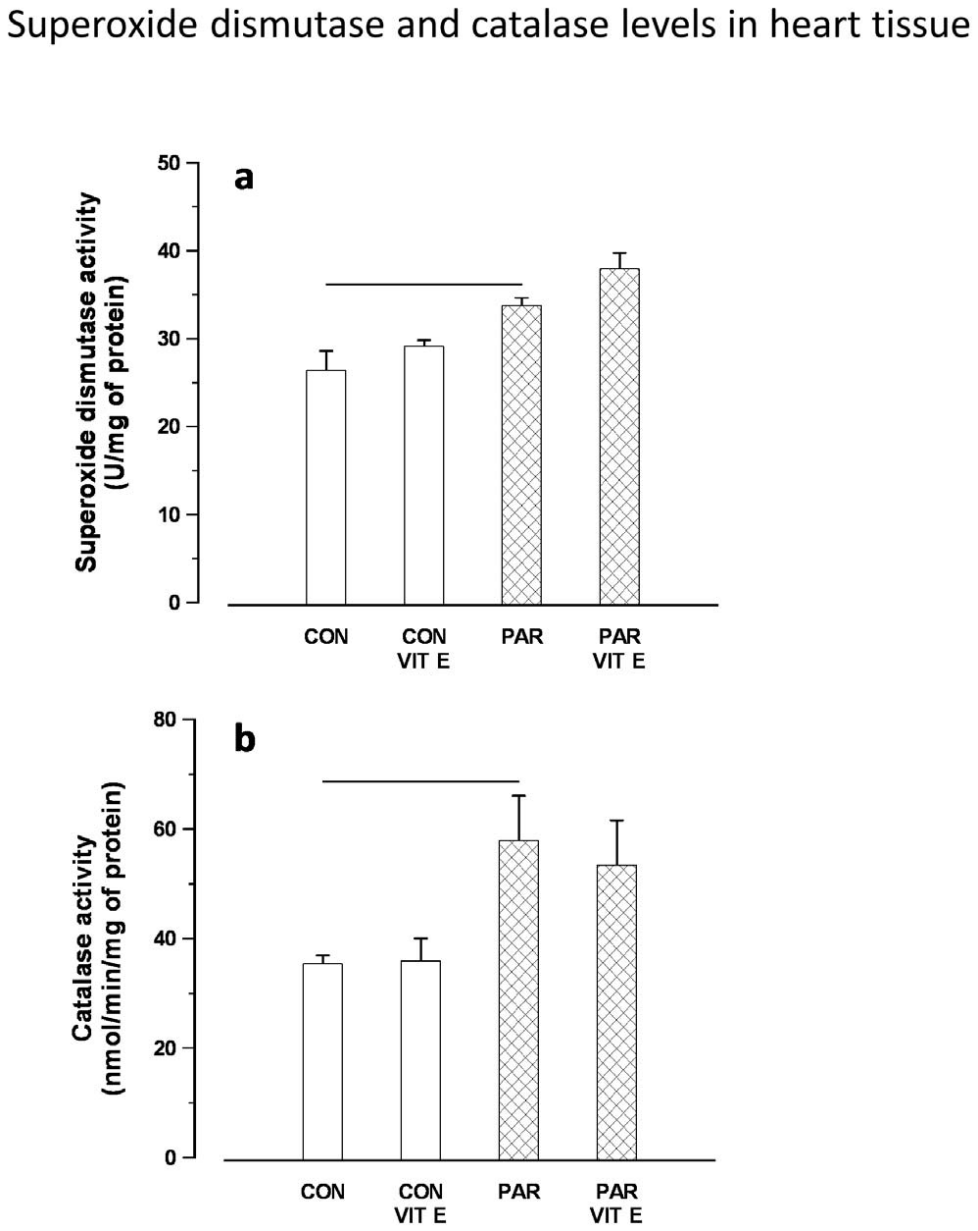
Superoxide dismutase (a) and catalse (b) levels in heart tissues of control and PAR exposed rats with or without vitamin E pretreatment. Data are mean ± SEM (n = 6). CON = Control, PAR = Paraquat, VIT E = Vitamin E. Horizontal lines above bars represent P<0.05.

Figure 5 illustrates the effect of PAR or saline with or without vitamin E pretreatment on the level of antioxidants in heart tissue including SOD and CAT. PAR caused a significant increase in SOD and CAT activities in heart tissue, indicating that PAR could initiate adaptive responses that counterbalance the potentially damaging activity of oxygen radicals induced by PAR exposure. SOD and CAT activities were increased in a comparable way in both PAR and vitamin E plus PAR groups.

## Discussion

The major findings of this study are: (1) Amplitude of shortening was reduced in myocytes from rats treated with PAR and unaltered in myocytes from rats treated with PAR and vitamin E compared to controls, (2) Amplitude of the Ca^2+^ transient was unaltered in myocytes from rats treated with PAR, (3) Myofilament sensitivity to Ca^2+^ was altered in myocytes from rats treated with PAR and unaltered in myocytes from rats treated with PAR and vitamin E compared to controls and (4) the antioxidants SOD and CAT enzymatic activities were significantly increased in heart tissue from rats treated with PAR compared to controls. These results suggest that PAR treatment reduces contractility of the heart and alters myofilaments sensitivity to Ca^2+^ and that these changes do not occur in the presence of vitamin E treatment. The higher levels of SOD and CAT enzymes may reflect a necessary response to face the damaging effect of oxidative stress due to PAR toxicity.

Although the current findings do not fully elucidate a mechanism of action for PAR, disturbances of mitochondrial function, generation of oxidative radicals and promotion of apoptotic factors are probably involved in decreased myocyte function [21]–[24].

Mitochondria are considered to be the major source of ROS in cells. Components of the electron transport chain are known to undergo auto-oxidation and generate ROS in the mitochondria. ROS generated in the mitochondria include superoxide anions, and hydroxyl radicals, among others. Hydroxyl radicals in ROS can injure cellular components, including DNA, and can result in extensive destruction to the cell.

Although low and intermediary levels of ROS are physiologically important, high ROS concentrations above the clearance capacity of the cell cause oxidative stress. Lipid peroxidation is one of the major outcomes of free-radical-mediated injury leading to mitochondrial dysfunction, cellular damage and, in many cases, cell death (15,16). Given the well-known capacity of PAR to promote generation of superoxide anion and other ROS [26] ROS accumulation and subsequent development of oxidative stress are perceived to be major factors underlying cardiac geometric and contractile alterations following PAR challenge. ROS is known to stimulate myocardial growth, matrix remodeling and cellular dysfunction.

PAR, a controversial herbicide, is one of the most widely used pesticides in the world. It is widely believed that PAR preferentially accumulates in the lung and exerts its cytotoxic effects via the generation of ROS and hence, many studies have focused on increasing the antioxidant status to counteract PAR-induced cytotoxicity [27].

Previous findings suggest that PAR exposure damages both dopaminergic cell bodies in the substantia nigra and nerve terminals in the striatum, but that compensatory mechanisms develop to allow for the maintenance of normal dopaminergic function. These mechanisms may include PAR-induced changes in phosphorylation or glycosylation of the receptor. PAR is thought to induce its toxic effects on the nigrostriatal system through its pro-oxidant actions, with the generation of large quantities of ROS [15]–[16].

Although in the acute and subacute phase of PAR poisoning many patients may die because of irreversible circulatory shock [28], [29], the effects of PAR on cardiac hemodynamics are not well understood. Various mechanisms have been suggested for oxidative stress-induced contractile depression including defects in receptor function [30], calcium pump and channel functions [31], [32] or, as noted below, disturbances in cardiac excitation-contraction coupling. Given the well known capacity of PAR to promote generation of superoxide anion and other ROS [18], ROS accumulation and subsequent development of oxidative stress are perceived to be major causes of cardiac dysfunction after PAR challenge.

Myofilament sensitivity to Ca^2+^ was assessed from phase-plane diagrams of fura-2 ratio versus cell length by measuring the gradient of the fura-2–cell length trajectory during late relaxation of the twitch contraction. The position of the trajectory during the relaxation phase reflects the relative myofilament response to Ca^2+^ and, hence, can be used as a measure of myofilament sensitivity to Ca^2+^ [24]. The gradient of the trajectory during the relaxation phase of the twitch contraction was significantly steeper in myocytes from PAR treated rats compared to controls suggesting altered myofilament sensitivity to Ca^2+^. Interestingly, myofilament sensitivity to Ca^2+^ was unaltered in myocytes from rats treated with PAR and vitamin E.

The endoplasmic reticulum(ER) is an extensive intracellular membranous network, a multifunctional organelle, that plays a central role in many essential cellular activities, such as folding, assembly and quality control of secretory and membrane proteins, disulfide bond formation, glycosylation, lipid biosynthesis, Ca^2+^ storage and signaling. Under stress conditions, such as perturbed Ca^2+^ homeostasis or redox status, elevated secretory protein synthesis rates, altered glycosylation levels, and hypercholesterolemia, unfolded or misfolded proteins accumulate in the ER lumen leading to ER stress. Unresolved and prolonged ER stress leads to perturbed Ca^2+^ homeostasis, increased protein accumulation, loss of ER function, and activation of apoptotic cascades [33].

Perturbation of ER Ca^2+^ homeostasis, a trigger for the accumulation of unfolded or misfolded proteins and activation of the ER stress response, seems to play an important role in the onset or progression of neuronal dysfunction [33]. ER directly communicates with mitochondria through close contacts referred as mitochondria-associated membranes that promote Ca^2+^ transfer from ER to mitochondria thus maintaining mitochondrial metabolism and cell survival [34]. Disruption of contact sites and impairment of Ca^2+^ coupling between ER and mitochondria have profound consequences for cellular function and in extreme cases lead to apoptosis.. Mitochondria and the ER are closely linked morphologically and functionally, and considerable crosstalk of cell death proteins, promoted by ROS and high Ca^2+^ levels, occurs between these two organelles. The Ca^2+^ transport systems of the ER are also sensitive to oxidative stress being directly exposed to ER/mitochondria-generated ROS. The resulting abnormal cellular Ca^2+^ load can trigger cell death by activating proteases, reinforcing signals leading to caspase activation, such as cytochrome c release from mitochondria, or by triggering other catabolic processes mediated by lipases and nucleases. In the present results, PAR was shown to promote excessive intracellular Ca^2+^ that could be released from ER, leading to overload of, and damage to mitochondria, therefore impaired Ca^2+^ homeostasis which may play a role in ROS generation, thereby disturbing cardiac function [40].

Skeletal *dorsiflexor* muscle contraction was investigated by measuring isometric twitch tensions (evoked either directly by stimulation of the muscle or indirectly by stimulation of motor nerve). Twitch tensions were recorded after the tendinous insertions were attached to a force displacement transducer. Isometric force of contraction in response to indirect supramaximal nerve and direct muscle stimulation were reduced in PAR treated animals [35]. The decremental effect of PAR treatment on both cardiac and skeletal muscles is interesting considering the differing mechanisms of muscle contraction. For example, cardiac muscle possesses gap junctions which allow coupling of neighboring cells while skeletal muscle fibers are insulated from each other. Control of sarcoplasmic reticulum (SR) Ca^2+^ release is voltage driven in skeletal muscle, however, L-type Ca^2+^ current is the primary trigger for SR Ca^2+^ release in cardiac muscle. While additional experimental work is needed to further clarify the exact mechanisms underlying the observed contractile decrement in cardiac and skeletal muscles, our findings corroborate previously published clinical studies which have described PAR-induced lesions in skeletal muscle [36], [37] and the heart [38]. Moreover, two cases of suicide from oral ingestion of PAR have been reported [39]. A histological examination revealed a marked degeneration of slow skeletal muscle fibers with an abrupt increase of plasma creatine kinase levels on the fifth day after PAR ingestion. The degeneration of the muscle was attributed to the long retention of PAR in the tissue [39].

PAR has been shown to act via a mechanism involving mitochondrial ROS production **leading to** extensive mitochondrial oxidative damage [41]. Elevated ROS levels in conjunction with increased Ca^2+^ levels are known promoters of programmed cell death [42] a fact that is consistent with the elevation of resting intracellular Ca^2+^ in myocytes from PAR treated rats and unaltered resting Ca^2+^ in myocytes from rats treated with PAR and vitamin E compared to controls.

Enzymatic defense against ROS involves the cooperative action of several antioxidant enzymes. SOD is one of the key enzymes that reacts with O_2_ to generate H_2_O_2_, which acts as a substrate for GPX and CAT to form H_2_O [43]. Our data show a significant increase of SOD and CAT levels in heart tissue. Superoxide dismutase and catalase levels were increased in an analogous manner in both PAR and vitamin E plus PAR groups. We suggest that the increase in oxidative stress caused by PAR [44], [45] was accompanied by increased antioxidant capacity, indicating that PAR could trigger adaptive responses that counterbalance the potentially damaging activity of oxygen radicals and limiting further oxidant-mediated lung inflammation [4], [5]. An increase in antioxidant activity has been reported in mice exposed to particulate air pollution or cigarette smoke [45], [46]. During PAR exposure cells continually suffer from oxidative stress in spite of over activity of the antioxidant defense mechanisms as indicated by increased SOD and CAT activity period the higher levels of antioxidants enzymes may be necessary to detoxify increased concentrations of lipid peroxidation products that are generated from oxidative stress due to PAR toxicity.

It can be concluded that heart function is compromised in rats exposed to PAR and that the abnormalities are ameliorated by vitamin E treatment. Moreover, PAR might initiate adaptive responses that counterbalance the potentially damaging activity of oxygen free radicals induced by PAR exposure. The higher levels of SOD and CAT may be a necessary measure to detoxify the increased level of oxidative stress due to PAR toxicity.
